# Zinc Regulates the Acute Phase Response and Serum Amyloid A Production in Response to Sepsis through JAK-STAT3 Signaling

**DOI:** 10.1371/journal.pone.0094934

**Published:** 2014-04-14

**Authors:** Ming-Jie Liu, Shengying Bao, Jessica R. Napolitano, Dara L. Burris, Lianbo Yu, Susheela Tridandapani, Daren L. Knoell

**Affiliations:** 1 Dorothy M. Davis Heart and Lung Research Institute, The Ohio State University, Columbus, Ohio, United States of America; 2 Center for Biostatistics, The Ohio State University, Columbus, Ohio, United States of America; 3 Department of Pharmacy, The Ohio State University, Columbus, Ohio, United States of America; French National Centre for Scientific Research, France

## Abstract

Sepsis rapidly activates the host inflammatory response and acute phase response. Severe sepsis, complicated by multiple organ failure, is associated with overwhelming inflammation and high mortality. We previously observed that zinc (Zn) deficiency significantly increases mortality in a mouse model of polymicrobial sepsis due to over-activation of the inflammatory response. In order to identify potential mechanisms that account for Zn-responsive effects, we generated whole exome expression profiles from the lung tissue of septic mice that were maintained on Zn modified diets. Based on systems analysis, we observed that Zn deficiency enhances the acute phase response and particularly the JAK-STAT3 pathway, resulting in increased serum amyloid A production. *In vitro* studies of primary hepatocytes and HepG2 cells substantiated that Zn-deficiency augments serum amyloid A production through up-regulation of the JAK-STAT3 and NF-κB pathways. In contrast, Zn inhibited STAT3 activation through the up-regulation of SHP1 activity. Collectively, these findings demonstrate that Zn deficiency enhances the acute phase response through up-regulation of the JAK-STAT3 pathway, thereby perpetuating increased inflammation that may lead to increased morbidity and mortality in response to sepsis.

## Introduction

Sepsis, a complex inflammatory syndrome that results from the host response to infection, is the leading cause of mortality in intensive care units in the U.S. [1]. Mortality from sepsis often occurs when the host response to systemic infection becomes dysregulated and over-amplified, resulting in severe sepsis, septic shock, and multiple organ dysfunction syndrome. The pathophysiology of sepsis is complex, which has so far limited our capacity to develop effective drug therapies [2]. Sepsis patients with a poor prognosis often exhibit over-activation of the initial innate immune response, which is accompanied by a markedly elevated cytokine response, also referred to as the cytokine storm [3].

During the initial stage of sepsis, the rapid release of cytokines, that include TNFα, IL-1β, and IL-6, rapidly activate the acute phase response (APR) primarily in the liver leading to the production of acute phase proteins (APPs) [4], [5]. IL-6 is widely viewed as the central mediator of APP production by hepatocytes [6]. In particular, IL-6 binds to its cognate membrane-bound gp130 receptor which leads to activation of the JAK-STAT3 and MAPK signaling pathways, resulting in the transcription of APPs. Inflammatory cytokines such as TNF and IL-1β are also essential for production of APPs, indicating the involvement of NF-κB signaling in production of APPs [7]. The fundamental role of APPs in the setting of sepsis remains poorly defined in part because APPs exhibit both pro- and anti- inflammatory functions [6], [8]. The over-production of APPs associated with overwhelming inflammation in sepsis is believed to be harmful [9]. However, impairment of the APR, which is recapitulated in IL-6, gp130, and RelA/STAT3 knockout mice, also exhibit worse outcomes in response to infection [7], [10], [11]. Collectively, this indicates that a critical balance in APP production is required to optimize host defense. More recently, APPs have been recognized as functional components of the innate immune response. For example, serum amyloid A (SAA), a family of 12–14 kDa apolipoproteins that are found predominantly in the high density lipoprotein (HDL) fraction of plasma, is now recognized as a potent opsonin [12] and activator of neutrophils [13]. SAAs are also involved in the recruitment and activation of leukocytes during the APR [11]. In particular, SAAs can induce the expression of pro-IL-1β through interaction with TLR2 and TLR4 which then activates the NLRP3 inflammasome [13], [14], [15].

Zn blood levels decline rapidly at the onset of the APR, which is referred to as hypozincemia, following its redistribution from the plasma into tissues [16]. A major proportion of Zn is redistributed into the liver following IL6-mediated induction of the Zn transporter ZIP14 [17]. The presumed beneficial role of hypozincemia is postulated to be akin to that of iron (hypoferremia), resulting in the strategic sequestration of bioavailable Zn from pathogenic microorganisms [18], [19]. Further, mobilization of Zn into the cellular compartment is required to facilitate gene transcription and protein production that includes the synthesis of APPs [20]. Zn also assists in the regulation of intracellular redox balance thereby playing a direct role in cytoprotection during the host response [21]. Related to this, our group has discovered that transport of Zn through the Zn transporter ZIP8 into the cytosol functions to inhibit IKKβ in monocytes and lung tissue, thereby controlling the extent of NF-κB activation and inflammation in response to sepsis [22], [23].

We previously observed that Zn deficiency significantly increases the systemic inflammatory response and mortality in a mouse model of polymicrobial sepsis [24]. Despite recent advances that involve the modulatory function of Zn on NF-κB [22], [23], much still remains unknown regarding the roles of Zn relative to the APR in the setting of sepsis. This is further complicated by the fact that Zn has been predicted to have direct involvement in up to 10% of the mammalian proteome [25]. Therefore, a systems-based approach involving genome-wide microarray analysis was used to reveal candidate genes and signaling pathways that account for the perturbations in host defense and survival in Zn-deficient, septic mice [24]. Strikingly, we revealed that the APR was significantly up-regulated in the setting of Zn deficiency in liver, accompanied by enhanced JAK-STAT3 signaling and a corresponding increase in SAA1 production. Importantly, Zn addition reduced JAK-STAT3 signaling and APR activity, indicating that it plays a pivotal role in balancing the initial host response through the APR in response to severe infection.

## Materials and Methods

### Animal studies

#### Establishing a mouse model of Zn-deficiency

Ten-week old, adult, male, C57BL/6 mice (∼25 g) (Harlan Sprague Dawley, Inc.) with fully developed lungs, were randomly placed on a Zn-deficient diet (Harlan Teklad, TD85419; Zn content: 0.5–1.5 ppm) or a matched control diet (TD85420; Zn content: 50 ppm) for three weeks, a time sufficient to establish subacute Zn-deficiency without requiring pair-feeding. A Zn-free environment was carefully maintained using deionized water in Zn-free containers and stainless steel cages. An additional group received a Zn-fortified diet (TD07129; 100 ppm) for three days following an 18 day Zn-deficient regimen [24].

#### Cecal ligation and puncture (CLP)

At the end of the dietary regime, mice were subject to cecal ligation and puncture (CLP) via laparotomy under general anesthesia (1% isofluorane) as described previously with slight modifications [24]. Briefly, through an upper midline abdominal incision, the cecum was delivered, ligated with a silk suture 1 cm from the tip, and doubly punctured with a 21-gauge needle. After puncture, the cecum was gently squeezed to extrude fecal content and returned to the abdominal cavity. The laparotomy was then closed. At 2, 4 or 24 hrs after CLP, the mice were sacrificed and blood and tissue samples were collected. Lung tissue was lavaged and perfused with saline prior to removal. Animal studies were approved by the Ohio State University Institutional Animal Care and Use Committee.

### Cell culture

Human hepatocellular carcinoma HepG2 cells (ATCC) were maintained in DMEM/F-12 with GlutaMAX supplemented with 10% FBS, 0.1 mg/mL streptomycin, 100 IU/mL penicillin, at 37°C in a 5% CO_2_ humidified atmosphere. Mouse primary hepatocytes were isolated from the livers of adult male C57BL/6 mice following collagenase perfusion. The abdominal inferior vena cava of the liver was cannulated. The hepatic portal vein was then cut through and the thoracic inferior vena cava was occluded with forceps. The liver was perfused with Hanks' balanced salt solution (HBSS, without calcium and magnesium) at 37°C for 5 min, followed by the perfusion with a collagenase buffer (0.5 mg/mL in HBSS) at 37°C for 10 minutes. After perfusion, the liver was rapidly excised and transferred to a sterile Petri dish. The cells were released by disrupting the liver capsule mechanically in William's E medium. Then the cells were separated from undigested tissue with a sterile 70-µm mesh nylon filter. After washing through low-speed centrifugation at 50×g for multiple times, cells were seeded and their viability were determined by counting using Trypan blue exclusion. The primary hepatocytes were cultured in William's E Medium with a specific supplemental cocktail (Invitrogen).

### RNA Extraction and quantitative RT-PCR

Total RNA was isolated from tissue or cells using TRIZOL reagent (Invitrogen). The cDNA synthesis was performed using ThermoScript RT-PCR System for First-Strand cDNA Synthesis (Invitrogen). Real-time PCR was performed with the 7900HT Real-Time PCR system (Applied Biosystems) using SYBR Green reagents. All analysis was normalized against the cycle threshold number of GAPDH or cyclophilin genes, then calculated using the following equation: RCN  =  *2* ^−ΔCt^ ×100, where ΔCt is the Ct_(target)_ – Ct_(reference)_. The sequences of all the probes are available upon request.

### Microarray analysis

Lung tissue RNA quality was first verified using the Bio-Rad Experion automated gel electrophoresis system (Bio-Rad). Microarray analysis was then conducted on individual lung RNA samples from control (Ctrl), Zn deficient (Zn-), control septic (Ctrl/CLP), Zn deficient septic (Zn-/CLP) and Zn supplemented septic (Zn+/CLP) mice (n = 3 per group). The Zn+/CLP group first received the Zn deficient diet for 18 days, a time sufficient to decrease plasma Zn levels greater than two-fold following administration of a Zn fortified chow for 3 additional days, a time sufficient to normalize plasma Zn levels. The Affymetrix-GeneChip Mouse Exon 1.0 ST array, which contains 193,387 probe sets that span 23,214 genes was used in our analysis. Affymetrix Expression Console Software was used to perform quality assessment including data filtration and normalization. Subsequent statistical analysis was performed using Partek Genomics Suite (PGS, Version 6.6, Partek Inc.).

### Pathway and network analysis

In order to identify alteration in major biologic pathways following different treatments, functional categorization and pathway construction was performed using Ingenuity Pathway Analysis (IPA) software (Ingenuity Systems, Inc.). Pair comparison gene lists were generated by PGS and then uploaded directly into IPA for analysis. The functional pathways or networks with the highest confidence scores were then determined. Corresponding scores for individual pathway and networks were obtained through comparative calculations obtained from the IPA system.

### Western blot analysis

Cell or tissue lysates were obtained with standard lysis buffer (20 mM Tris-HCl (pH 7.5), 150 mM NaCl, 1 mM Na_2_EDTA, 1 mM EGTA, 1% Triton X-100, 2.5 mM sodium pyrophosphate, 1 mM β-glycerophosphate, 1 mM Na_3_VO_4_, 1 µg/mL leupeptin, 1 mM PMSF) containing protease inhibitors (complete protease inhibitor cocktail (Roche)). The proteins were separated by SDS-PAGE and transferred to nitrocellulose membranes. The membranes were blocked with 5% skim milk in TBST, followed by probing with antibodies overnight. All the antibodies were purchased from Cell Signaling Technology, Inc. The chemiluminescent signal was detected using Amersham ECL reagents (GE Healthcare).

### Serum amyloid A (SAA) determination

The quantification of serum amyloid A protein levels in cell culture supernatants was conducted after exposure to IL-1 + IL-6 alone or in combination with the Zn chelating agent TPEN or Zn + pyrithione. Mouse serum was separated from whole blood by centrifugation at 1000×g for 10 minutes at 4°C. Quantification of SAA1 levels was determined by ELISA according to the manufacturer's instructions (Invitrogen).

### SHP1 phosphatase activity assay

The SHP-1 phosphatase activity in HepG2 cells was determined using a SHP-1 immunoprecipitation (IP) based specific assay (DuoSet IC, R&D Systems) according to manufacturer's instructions. Briefly, immunoprecipitation was performed using an anti-SHP-1 antibody conjugated to agarose beads. After washing, the beads were incubated with a synthetic phosphopeptide substrate that is subject to dephosphorylation by SHP-1 resulting in release of free phosphate. The amount of free phosphate was then determined by a sensitive dye-binding assay using malachite green and molybdic acid. The total amount of SHP-1 following immunoprecipitation was determined by Western analysis and subsequently used for data normalization.

### Zn measurement by atomic absorption spectroscopy (AAS)

For atomic absorption spectroscopy, HepG2 cells were washed at 90% confluence twice with equal volumes of PBS. The cells were harvested in 1% SDS-lysis buffer, followed by digestion with a mixed acid solution of nitric acid: perchloric acid (1∶2) at 80°C for 4–6 hrs. Acid-digested samples were diluted with MilliQ water and then measured using an AAnalyst 400 spectrophotometer (Perkin Elmer). The total protein content was determined using the BCA assay. The final values were normalized to total cellular protein content.

### Statistical analysis

All data are presented as mean ± SE or SD. For comparison between multiple groups, a one-way or two-way ANOVA with post-hoc test, such as Tukey's or Bonferroni test, was used. A Student t-test was used for comparison between two groups. Statistical significance was defined at p-value of less than 0.05 (p<0.05).

## Results

### Microarray analysis reveals a Zn-responsive gene cluster in the setting of sepsis

**Figure 1:**
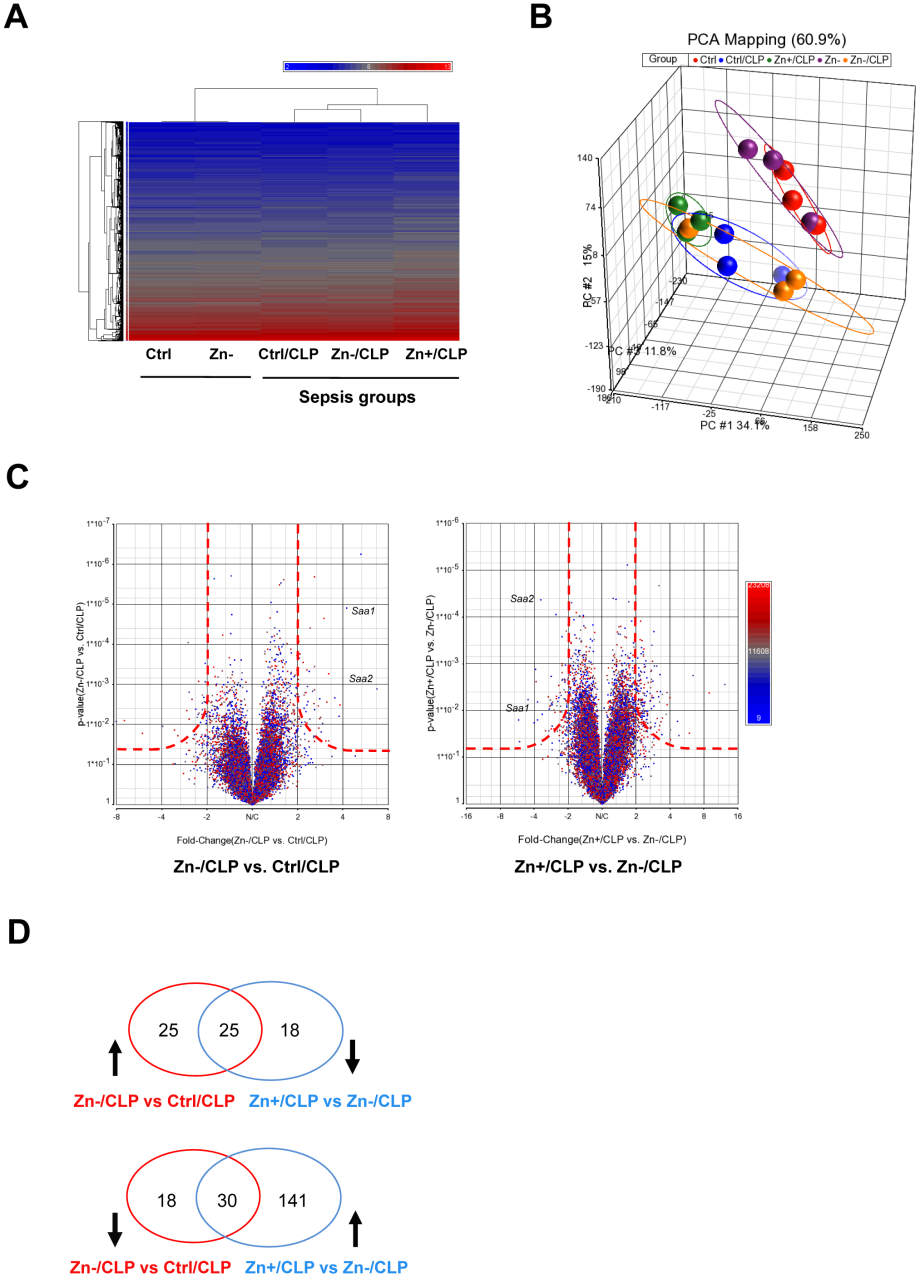
Genome profiling of mouse lung tissue in the combined setting of cecal ligation and puncture (CLP)-induced sepsis and modified Zn diets. (A) A two-dimensional gene cluster map is shown representing the entire genome (>20,000 genes) that are differentially regulated between Ctrl, Zn- (Zn deficiency), CLP alone (Ctrl/CLP), Zn deficiency with CLP (Zn-/CLP), and Zn supplementation with CLP (Zn+/CLP) treatment groups (The mean values are expressed as the average from 3 mice per group for heatmap analysis). C57/B6 mice were administered a control diet (Ctrl), Zn-deficient (Zn-) diet, or a Zn-deficient diet for 18 days followed by oral Zn-supplementation (Zn+) diet for 3 more days. The three week dietary regimes were then followed by CLP and tissue harvest at 24 hrs post CLP and then microarray analysis. (B) Principal component analysis revealed distinct relationships between different groups. CLP alone resulted in a significant change in global gene expression, compared to non-CLP groups, which was further influenced by Zn status. (C) The adjacent volcano plots illustrate that multiple genes are differentially influenced by Zn status and that Saa1 and Saa2 are two of the most “Zn responsive” genes in the setting of CLP. The cut-off boundary is shown as a red dash line. (D) The Venn diagram summarizes the number of “Zn-responsive” genes in the setting of CLP. Specifically, the number of “Zn-responsive genes” is shown at the intersection generated when we compared different treatment groups as already described.

Zn deficiency increases pre-existing systemic inflammation, vital organ damage and mortality in response to polymicrobial sepsis [23], [24]. In order to reveal potential underlying mechanisms that account for the effects of Zn, we performed a comprehensive microarray experiment on mouse lung tissue and compared the gene expression profiles between different treatment groups. Hierarchical cluster analysis was first conducted. CLP-treated mice were distinctly different when compared to untreated mice (Ctrl and Zn-) (Fig. 1A). This was further substantiated by principle component analysis (PCA) (Fig. 1B). Somewhat surprising, no substantial changes were observed when we compared mice maintained only on control and Zn-deficient diets without CLP (Ctrl and Zn-). Next we performed pairwise comparisons to determine changes in gene expression profiles between different treatment groups. A volcano plot, which highlights fold change (log2) on the X-axis and the corresponding p-value (negative log10) on the Y-axis, was used to identify genes of interest. Again, CLP significantly induced changes in gene expression profiles (Fig. S1B). Zn deficiency alone did not significantly alter the profile compared to mice maintained on the control diet, with only three genes demonstrating significant change (*Cyp1a1*, *Fkbp5*, and *Angptl4*) (Fig. S1C). Importantly, Zn deficiency or Zn supplementation in combination with CLP substantially altered gene expression profiles when compared to the Ctrl/CLP treatment group (Fig. 1C), as shown in Table 1 and summarized by the Venn diagrams (Fig. 1D). The overlapping gene cluster in the Venn diagram designates all of the possible ‘Zn-responsive’ genes which emphasizes that the expression of each of these genes increased and decreased as a result of Zn intake. As a representative example, serum amyloid a1 and a2 (*Saa1* and *Saa2*) were both identified as Zn-responsive genes (Fig. 1C).

**Table 1:** The Zn-responsive gene list in the setting of CLP-induced sepsis.

Gene Symbol	RefSeq	fold change (Zn-/CLP vs. Ctrl/CLP)	p-value (Zn-/CLP vs. Ctrl/CLP)	fold change (Zn+/CLP vs. Zn-/CLP)	p-value (Zn+/CLP vs. Zn-/CLP)
***Saa1***	**NM_009117**	**6.7203**	**0.00131025**	**0.22161**	**0.00582652**
*Timp1*	NM_001044384	5.2676	5.68E-07	0.391976	8.90E-05
***Saa2***	**NM_011314**	**4.23408**	**1.26E-05**	**0.286493**	**4.33E-05**
*Slc39a14*	NM_144808	3.78351	0.00625132	0.267834	0.00662228
*Selp*	NM_011347	3.21295	0.0258389	0.316672	0.0276154
*Mt1*	NM_013602	3.1283	0.00277035	0.471856	0.0267018
*Serpine1*	NM_008871	2.93211	0.00948452	0.449371	0.0386225
*Bcl3*	NM_033601	2.84165	0.0066394	0.377024	0.00971195
*Socs3*	NM_007707	2.81513	0.00231141	0.462428	0.0128865
*Adamts4*	NM_172845	2.80698	0.00720106	0.333772	0.00504667
*Alpl*	NM_007431	2.75951	0.0124632	0.260677	0.00241287
*Mt2*	NM_008630	2.74561	0.00343479	0.393946	0.00560257
*Btla*	NM_001037719	2.57681	0.00304324	0.497342	0.0167992
*Cxcl14*	NM_019568	2.43534	3.94E-05	0.482897	0.000202528
*Mrps36*	NM_025369	2.37116	0.0266468	0.421509	0.0265732
*Porcn*	NM_016913	2.33058	0.00113282	0.463466	0.00216082
*E030010A14Rik*	NM_183160	2.30372	0.00913598	0.481085	0.0179898
*Tnip1*	NM_021327	2.30366	0.00329029	0.467629	0.00579137
*Treml4*	NM_001033922	2.25595	0.0416154	0.398957	0.0247957
*Tnfaip3*	NM_001166402	2.23892	0.00868978	0.478242	0.0138959
*Slc38a4*	NM_027052	2.20663	0.00187381	0.443617	0.0015678
*Sele*	NM_011345	2.09059	0.0333765	0.449022	0.0232268
*Jak3*	NM_010589	2.05812	0.00715066	0.477877	0.00627804
*Irf7*	NM_016850	2.03309	0.0064479	0.489741	0.00622596
*Midn*	NM_021565	2.00362	0.0147108	0.474885	0.010271
*1700094D03Rik*	NM_028567	0.492701	0.00941697	2.49419	0.00201898
*Col14a1*	NM_181277	0.48775	0.00274399	2.20433	0.00145418
*P2ry1*	NM_008772	0.48727	0.0181395	2.14769	0.0133659
*Sgcd*	NM_011891	0.484101	0.00417675	2.18139	0.00265518
*Fam55d*	BC094249	0.476895	0.00209652	2.02417	0.00287543
*Sdpr*	NM_138741	0.468204	0.0232725	2.7322	0.00532373
*Ecm2*	NM_001012324	0.466824	0.00430672	2.17353	0.00383651
*Aspn*	NM_025711	0.46532	0.00291191	2.76085	0.000406716
*1700009P17Rik*	BC061017	0.461606	0.00563526	2.04289	0.0088213
*BC028528*	BC028528	0.459351	0.0203715	2.75969	0.00490951
*Gja5*	NM_008121	0.454655	0.0204968	2.46549	0.0103728
*Cxx1c*	NM_028375	0.453209	0.00898742	2.29777	0.00679662
*Mfap5*	NM_015776	0.445323	0.0251303	2.5464	0.0124711
*Fam13a*	NM_153574	0.439517	0.000851912	2.33264	0.000687444
*Pcolce2*	NM_029620	0.437593	0.00571057	2.90089	0.00112144
*Vsnl1*	NM_012038	0.431588	0.00658066	2.7411	0.00214303
*Emcn*	NM_001163522	0.425039	0.017489	3.07106	0.00392945
*Slc5a12*	NM_001003915	0.421425	0.049591	2.95332	0.0188453
*Angpt1*	NM_009640	0.420946	0.0301067	3.22653	0.00655852
*Cyp2e1*	NM_021282	0.412813	0.0179354	2.73682	0.00921796
*Itga8*	NM_001001309	0.403547	0.0224958	3.41136	0.00450198
*Abi3bp*	NM_001014423	0.395998	0.00237429	2.70704	0.00146761
*1810011O10Rik*	NM_026931	0.382483	0.0443551	3.43976	0.0144159
*Scgb3a2*	NM_054038	0.37513	9.00094E-05	3.1996	2.15142E-05
*Scgb3a1*	NM_170727	0.366715	0.0286004	4.0147	0.00535128
*Gria1*	NM_008165	0.365633	0.00449927	2.23411	0.0155068
*Stmn2*	NM_025285	0.303428	0.0137715	3.78398	0.00765716
*Ogn*	NM_008760	0.253138	0.0110566	6.4349	0.00178623
*Car3*	NM_007606	0.141459	0.00811154	8.92868	0.00420472
*Plunc*	NM_011126	0.125153	0.00856262	12.27	0.00280282

### Zn modulates the acute phase response and STAT3 signaling pathways

**Figure 2:**
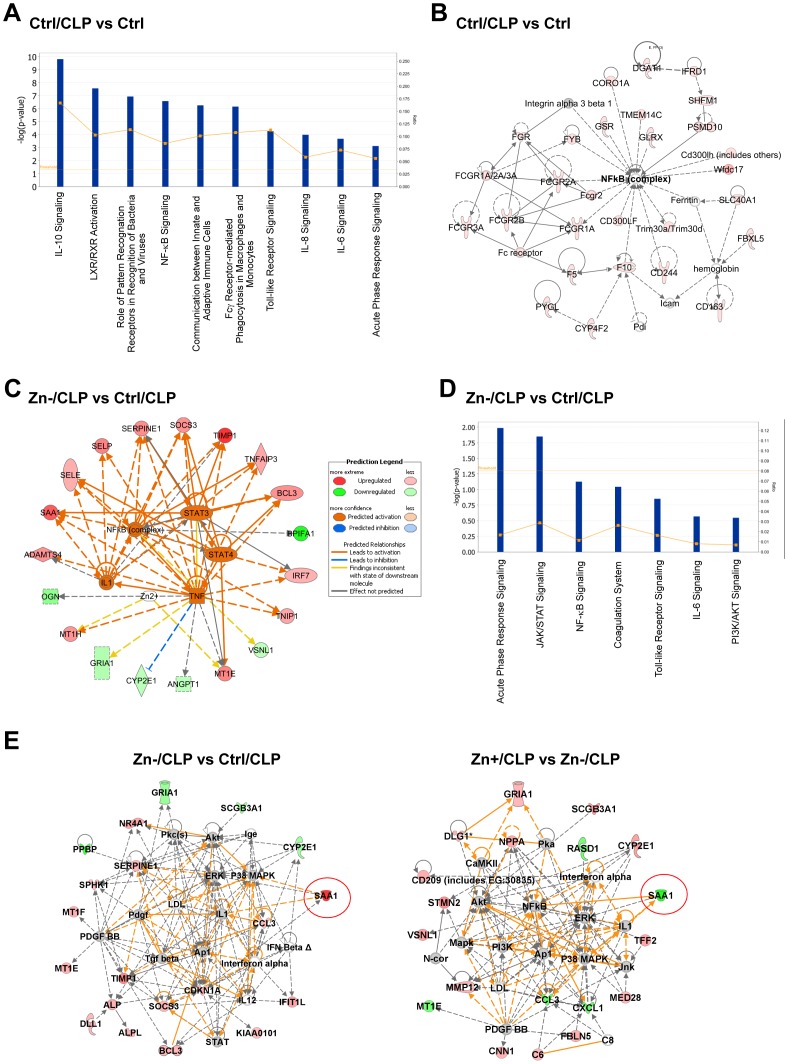
Pathway and network analysis of mouse lung in response to CLP and modified Zn diets. (A) A summary of the top 10 canonical pathways that are regulated by CLP alone. (**B**) One of the top networks identified among CLP-regulated genes. Red  =  up-regulated genes. (**C**) Pathway analysis identified a major regulome that predicts how Zn deficiency may regulate the response to CLP. (**D**) The major canonical pathways identified that are most affected by Zn deficiency in response to CLP. (**E**) A comparison between the effect of Zn deficient and Zn supplemented diets (both in comparison to Ctrl/CLP treatment) that identifies SAA1 as one of the most affected Zn-responsive genes.

To further understand the biological significance of expression profiling, we next compared the paired-comparison gene lists using Ingenuity Pathway Analysis [26]. Many of the canonical pro-inflammatory pathways were activated in response to CLP-induced sepsis, including cytokine signaling (IL-10, IL-8 and IL-6), the NF-κB pathway, the pattern recognition pathway (Toll-like receptors), and the acute phase response pathway (Fig. 2A). Network analysis also revealed the top gene networks affected by CLP-induced sepsis (Fig. 2B and Fig. S2A). Zn deficiency induced changes in specific gene clusters in the setting of CLP. Upstream transcriptional regulators (as part of the regulome) included NF-κB, TNF, IL1 and STAT3, highlighting modulation of these pathways by Zn (deficiency) (Fig. 2C). Regulome predictions were further confirmed by canonical pathway analysis. The top three pathways that were up-regulated by Zn deficiency were the acute phase response, JAK/STAT and NF-κB signaling pathways. We then analyzed the networks to identify significant functional modules that were perturbed in response to Zn intake (Fig. 2E and Fig. S2B). Our analysis revealed that Zn affects multiple pathways and networks in the setting of CLP-induced sepsis. Importantly, SAA1 was identified as one of the most robust Zn-responsive genes in which case Zn deficiency augmented its expression whereas Zn supplementation decreased its expression in response to sepsis.

### Zn deficiency augments acute phase response and STAT3 activation in septic mice

**Figure 3:**
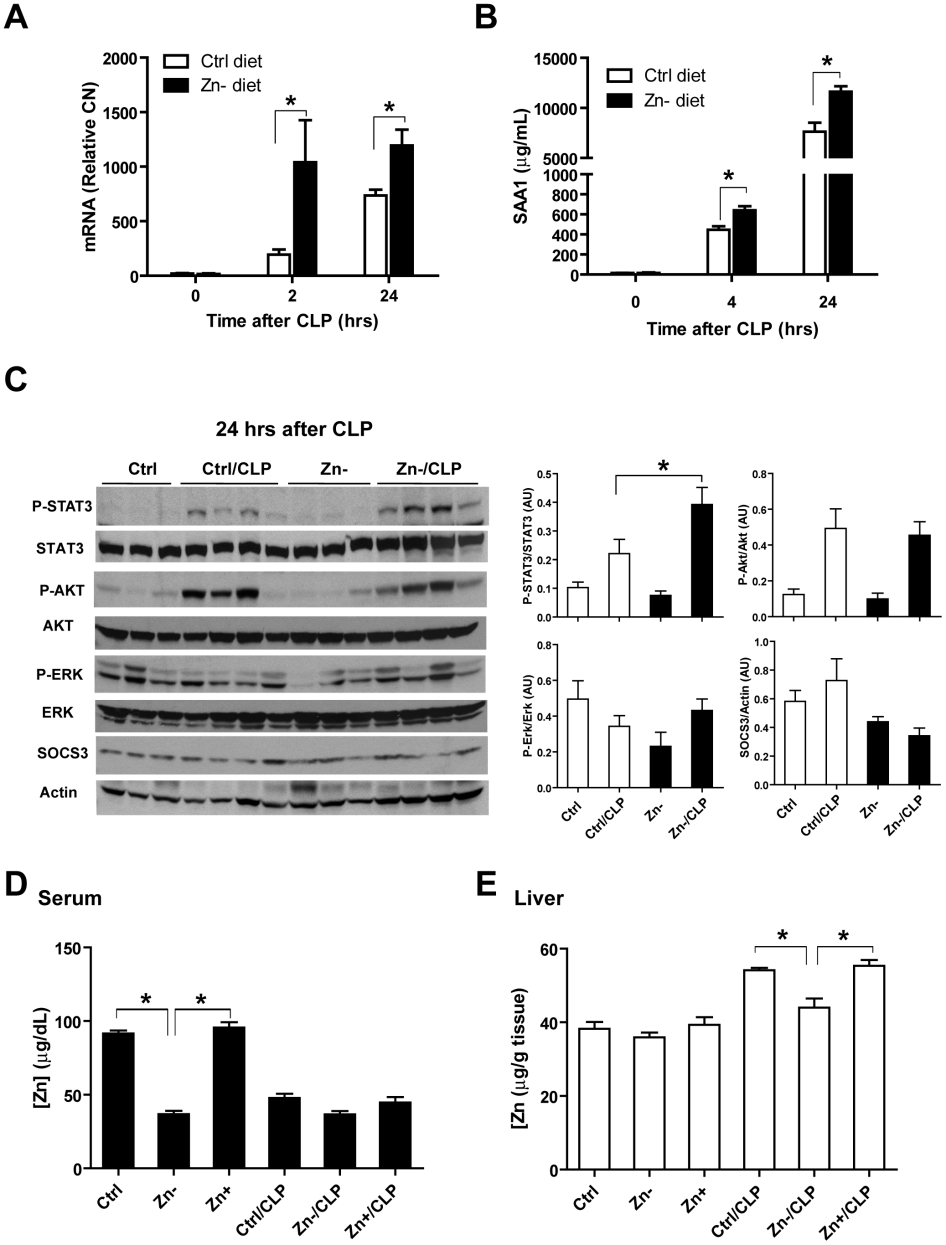
The acute phase response protein serum amyloid A1 (SAA1) is up-regulated by Zn deficiency in response to polymicrobial sepsis. (A) SAA1 mRNA analysis in the liver of mice in response to the combined treatment of Zn-deficiency and CLP compared to other treatment groups (n = 5 per treatment group). (* p<0.05, two-way ANOVA with Bonferroni post-hoc test) (B) Time-lapse analysis of serum SAA1 levels (n = 5 per treatment group). (* p<0.05, two-way ANOVA with Bonferroni post-hoc test) (C) Western analysis of the STAT3 pathway in the liver of the mice following Zn-modified diets and/or CLP treatment (n = 3-4 per treatment group) and corresponding densitometric analysis. AU: Arbitrary units (* p<0.05, 2×2 factorial ANOVA). Zn levels in serum (D) and liver (E) in response to Zn-modified diets and/or CLP treatment (n = 3–4 per treatment group) (* p<0.05, one-way ANOVA with Tukey's post-hoc test).

Knowing that acute phase proteins are mainly produced by the liver, we then analyzed liver tissue to determine whether the acute phase response and STAT3 signaling pathway were Zn-responsive in septic mice. SAA1 gene expression was significantly induced by CLP and further augmented by Zn deficiency at 2 hrs and 24 hrs (Fig. 3A). Consistent with this, serum SAA1 levels were also significantly increased by Zn deficiency at 4 and 24 hrs (Fig. 3B). At 24 hrs after CLP, Zn deficiency increased STAT3 phosphorylation (Fig. 3C). Collectively, these data support that Zn deficiency increases STAT3 activation in the liver of septic mice, resulting in enhanced production of the acute phase protein SAA1.

### Zn deficiency augments SAA1 production in mouse primary hepatocytes

**Figure 4:**
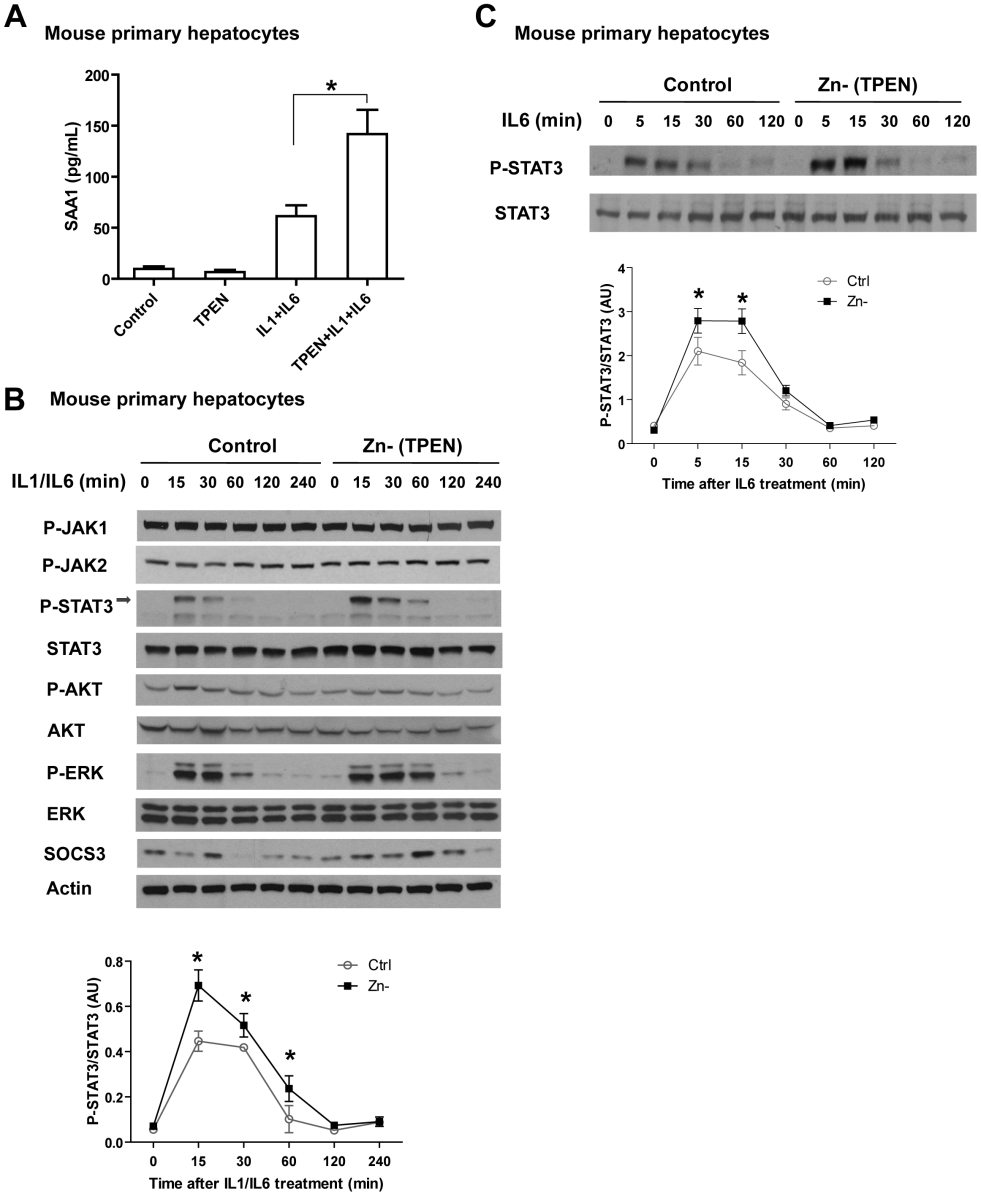
The effects of Zn deficiency on SAA1 production in response to IL-1 and IL-6 in mouse primary hepatocytes. (**A**) Zn deficiency, via TPEN chelation, increases SAA1 production in mouse primary hepatocytes (Error bars represent standard deviation. Data are representative of three independent experiments, * p<0.05, one-way ANOVA with Tukey's post-hoc test). (**B**) Zn deficiency increases STAT3 activation in response to IL-1 (20 ng/mL)/IL-6 (20 ng/mL) treatment. (**C**) Zn deficiency increases STAT3 activation in response to IL-6 alone. Densitometric analysis was conducted for panel B and C (Error bars represent standard deviation. Data are representative of three independent experiments. * p<0.05, two-way ANOVA with Bonferroni post-hoc test).

In order to better understand the impact of Zn deficiency on the APR *in vivo*, we prepared mouse primary hepatocytes and analyzed their response to IL-1/IL-6 in conjunction with TPEN (N,N,N′,N′-tetrakis(2-pyridylmethyl)ethylenediamine), a membrane permeable Zn chelator used to establish Zn deficiency in vitro. We observed that Zn deficiency significantly enhanced SAA1 release in response to IL-1/IL-6 when compared to Zn-sufficient cultures (Fig. 4A). We then examined candidate signaling pathways and observed that the phosphorylation of STAT3 and ERK was enhanced by Zn deficiency (Fig. 4B). In addition, augmentation of STAT3 phosphorylation by Zn deficiency also occurred in cells treated with IL-6 alone, further confirming that Zn deficiency modulates the IL-6-STAT3 pathway.

### Zn regulates SAA1 production through STAT3 and NF-κB signaling pathways

**Figure 5:**
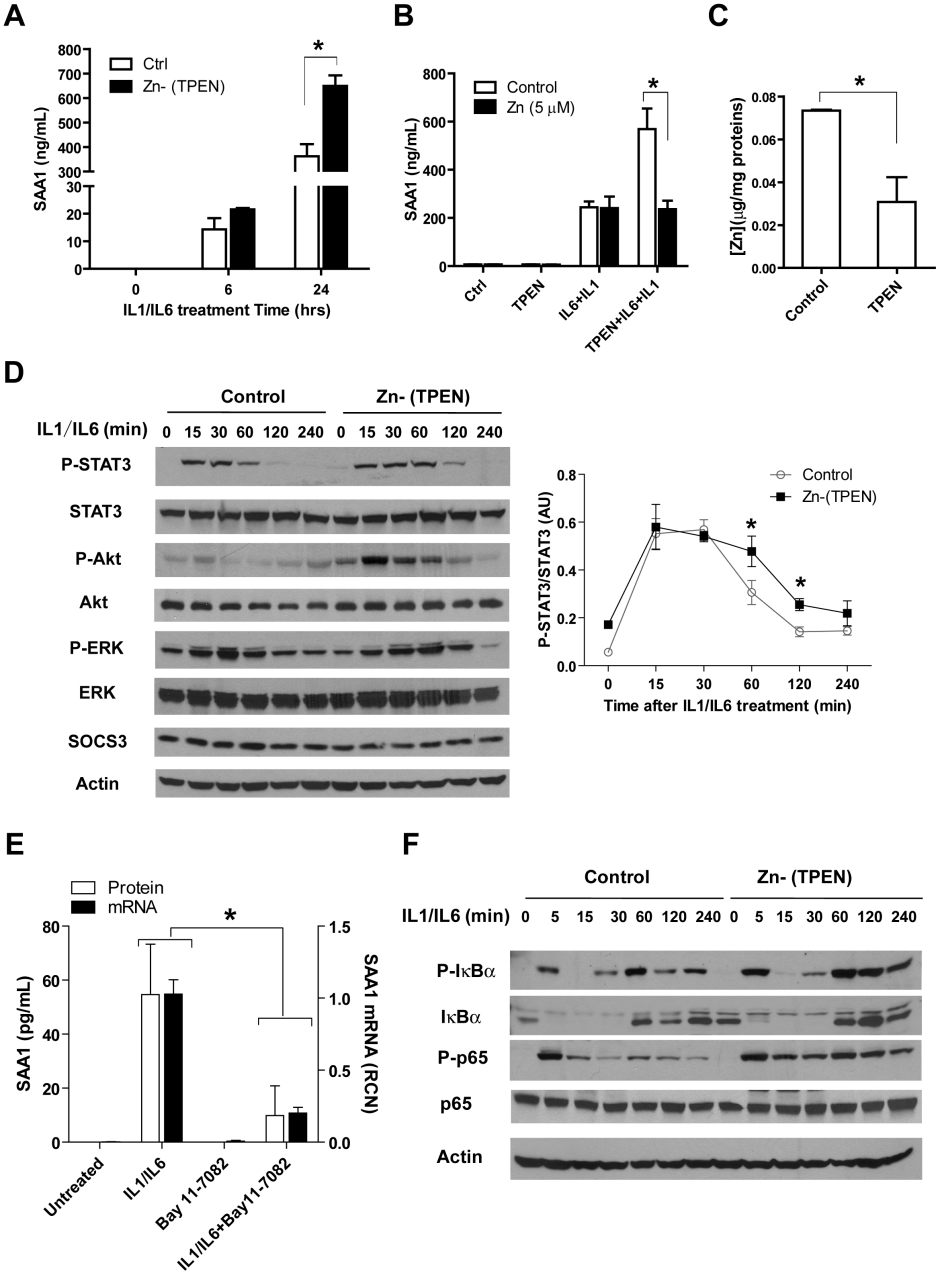
Zn deficiency increases STAT3 and NF-κB signaling in response to IL-1 and IL-6 in HepG2 cells. (**A**) SAA1 protein increases in the supernatant of HepG2 cell cultures in response to IL-1 (20 ng/mL) and IL-6 (20 ng/mL) treatment but more so when cultures were pretreated with TPEN (10 µM for 30 min). (**B**) Zn supplementation (5 µM ZnSO4) reversed the TPEN effect as exhibited by lower SAA1 production. ZnSO4 was added after TPEN treatment, but 5 min before IL-1/IL-6 treatment. (**C**) Intracellular Zn content is significantly reduced in HepG2 cells following TPEN treatment (10 µM, 30 min). (**D**) Analysis of candidate signaling pathways in response to IL-1/IL-6 in the presence or absence of TPEN. Densitometric analysis (pSTAT3/STAT3) was shown on the right. (**E**) The NF-κB inhibitor Bay 11-7082 significantly inhibits SAA1 expression and production induced by IL-1/IL-6. HepG2 cells were pretreated with Bay 11-7082 (5 µM, 30 min), followed by IL-1/IL-6 treatment. SAA1 mRNA analysis was performed at 2 hrs after IL-1/IL-6 treatment; SAA1 protein levels in the medium were detected at 24 hrs after IL-1/IL-6 treatment. (**F**) Alteration of NF-κB pathway signaling in response to the combined treatment of IL-1/IL-6 and TPEN. (Error bars represent standard deviation. Data are representative of three independent experiments, * p<0.05. A, B, and D, two-way ANOVA with Bonferroni post-hoc test; C, Student t-test; E, 2×2 factorial ANOVA)

In pursuit of the underlying mechanism(s) how Zn modulates the APR, we utilized the human liver cell line HepG2 and first confirmed the synergistic effect of Zn deficiency (TPEN) on SAA1 production in response to IL-1/IL-6 treatment (Fig. 5A). Further, augmentation of SAA1 production induced by IL-1/IL-6 combined with TPEN was normalized by the addition of Zn, indicating that this effect is Zn specific (Fig. 5B). Importantly, we confirmed that TPEN treatment did indeed significantly decrease intracellular Zn levels (Fig. 5C). As an alternative approach, we also exposed HepG2 cells to Zn-deficient medium (DMEM plus FBS treated with Chelex-100, a resin that depletes Zn) for an extended time [27]. Consistent with previous findings, SAA1 production was enhanced in cultures exposed to Zn devoid medium (Fig. S3A–B). Further, we then examined corresponding signaling pathways and observed that Zn deficiency increased STAT3 phosphorylation in HepG2 cells in response to IL-1/IL-6. Akt phosphorylation, which occurs downstream of STAT3, was also modulated by Zn status (Fig. 5D). Knowing that IL-1 activates the NF-κB and Zn interacts with NF-κB pathway, we then determined whether NF-κB also plays an essential role in the production of SAA1. We observed that the NF-κB specific inhibitor Bay 11-7082 significantly inhibited SAA1 expression and release, demonstrating that NF-κB plays a contributing role in SAA1 transcription (Fig. 5E). We further analyzed signaling events within the NF-κB pathway and observed that Zn deficiency increased IκBα and P65 phosphorylation (Fig. 5F), indicating that Zn modulates IKK complex activity, which is consistent with our previous findings [23].

### Zn inhibits STAT3 activation through modulation of SHP1activity in HepG2 cells

**Figure 6:**
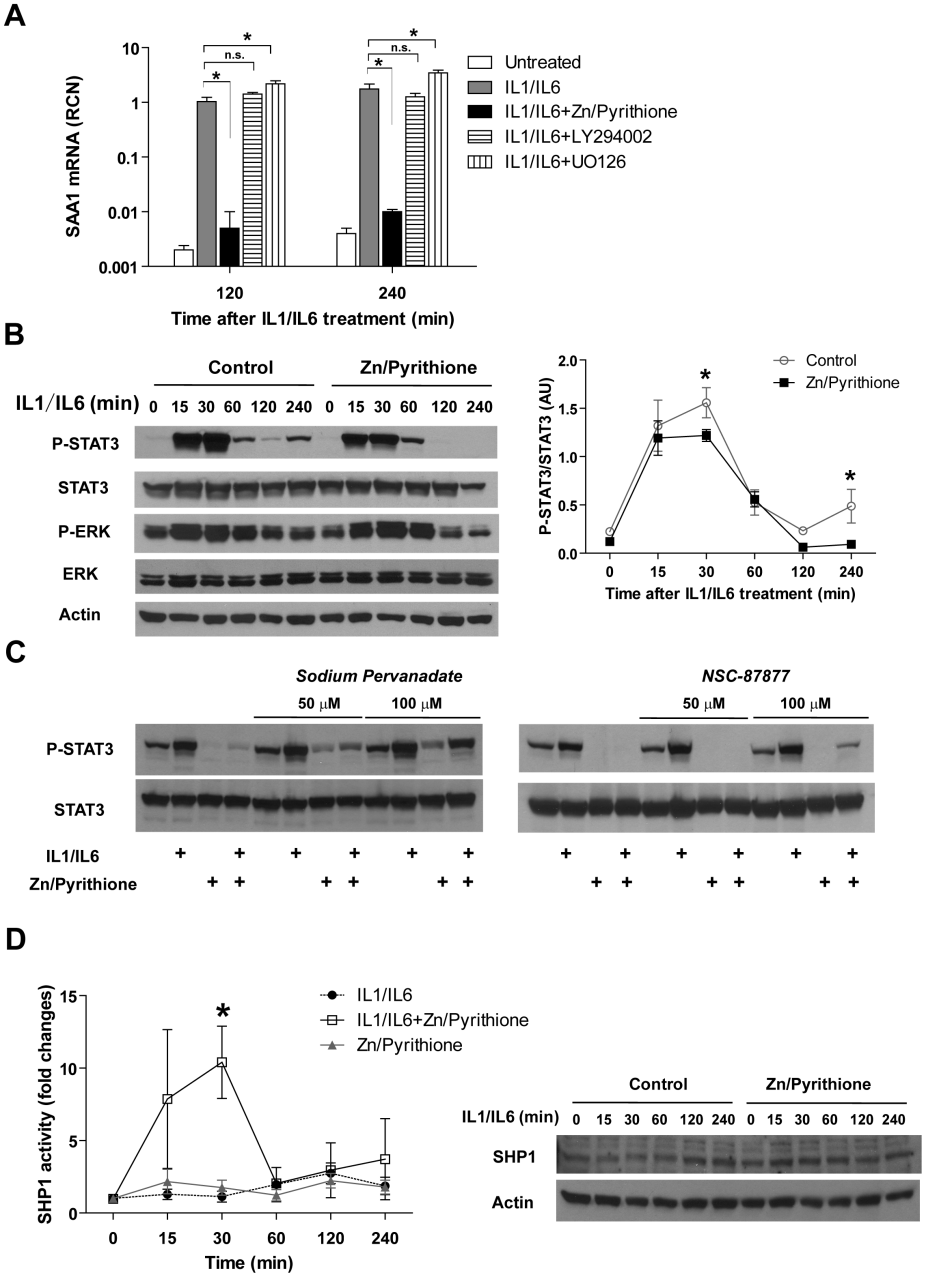
Zn inhibits STAT3 phosphorylation through up-regulation of SHP1 activity. (**A**) ZnSO4 (10 µM), together with its ionophore pyrithione (10 µM), inhibited SAA1 transcription in response to IL-1/IL-6 treatment in HepG2 cells. PI3K inhibitor LY294002 (10 µM) and ERK inhibitor UO126 (10 µM) did not inhibit or even increase SAA1 expression (Error bars represent standard deviation. Data are representative of three independent experiments. * p<0.05, Two-way ANOVA with Bonferroni post hoc). (**B**) Zn/pyrithione inhibits STAT3 phosphorylation in response to IL-1 (20 ng/mL)/IL-6 (20 ng/mL) treatment in HepG2 cells. Densitometric analysis is shown to the right (Error bars represent standard deviation. Data are representative of three independent experiments, * p<0.05. Two-way ANOVA with Bonferroni post hoc). (**C**) The nonspecific tyrosine phosphatase inhibitor sodium pervanadate and the SHP1-specific inhibitor NSC-87877 prevented the Zn/Pyrithione-induced inhibition of STAT3 phosphorylation at 4 hrs after IL-1/IL-6 treatment. (**D**) Zn augmented SHP1 activity at 30 min after IL-1/IL-6 treatment (Error bars represent standard deviation. Data are representative of three independent experiments, * p<0.05. Two-way ANOVA with Bonferroni post hoc). Western blot on the right shows the change in total SHP1 levels.

Having established that Zn deficiency increases STAT3 phosphorylation and SAA1 production, we next determined whether Zn inhibits SAA1 expression through the STAT3 pathway. HepG2 cultures were exposed to IL-1/IL-6 in the presence of Zn and the Zn ionophore pyrithione, which allows Zn to bypass transporter-mediated uptake and directly access the cytosol. Zn in combination with pyrithione significantly inhibited SAA1 transcription. At the same time, the inhibitors specific for PI3K and ERK kinases did not have a significant inhibitory impact on SAA1 transcription, indicating the effect of Zn/pyrithione is possibly not through these two pathways because PI3K-Akt and ERK pathways were not directly involved for SAA1 transcription (Fig. 6A). We further demonstrated that Zn/pyrithione inhibits STAT3 phosphorylation, especially at later times following IL-1/IL-6 treatment (Fig. 6B and Fig. S4). Knowing that STAT3 phosphorylation is also regulated by protein tyrosine phosphatases (PTPs), that include SHP1 and SHP2, we pretreated cultures with either the general phosphatase inhibitor sodium pervanadate or the SHP1/2 -specific inhibitor NSC-87877 under similar conditions. Both inhibitors modestly prevented the inhibition of STAT3 phosphorylation by Zn/pyrithione (Fig. 6C). This led us to postulate that the inhibitory effect of Zn/pyrithione on phospho-STAT3 may occur due to modulation of SHP1/2 activity. Using a specific immunoprecipitation-based SHP1 phosphatase assay, we measured the activity of SHP1 following IL-1/IL-6 treatment in the absence or presence of Zn/pyrithione. We observed that Zn/pyrithione significantly increased SHP1 activity at 30 min after IL-1/IL-6 treatment. Meanwhile, Zn/pyrithione did not influence the total protein levels of SHP1 (Fig. 6D). These findings demonstrate that SHP1 activity is altered in situ by Zn, thereby contributing to STAT3 inhibition. Exactly how Zn modulates SHP1 activity in cells will require further investigation.

## Discussion

Despite recent improvements in patient care, sepsis-related mortality still remains high [28]. This is in part due to the fact that we do not yet fully understand the pathogenesis of this syndrome, which is further complicated by an extremely heterogeneous patient population. Different approaches to inhibit inflammatory mediators including TNF, IL-1 and leukotrienes, have been clinically evaluated with discouraging results [28], [29]. Moreover, activated protein C, the only approved treatment in the United States, was recently withdrawn from the market [30]. The failure of multiple clinical trials re-emphasizes that further elucidation of the pathophysiologic mechanisms that account for sepsis should remain a high priority [28], [31].

Zn is the second most abundant trace element, next to iron, in our bodies and is required for proper immune function and defense against pathogens [32]. Nutritional deficits in Zn increase susceptibility to infection [33], [34], [35], whereas Zn supplementation can prevent or decrease the extent of infection [35], [36], [37]. The magnitude of the inflammatory response that immediately occurs following systemic infection is inversely correlated with Zn plasma levels [38], [39]. In particular, lower plasma Zn levels are associated with increased injury to vital organs and higher mortality [38], [40]. Upon pathogen invasion, Zn rapidly redistributes from the blood compartment to other tissues, particularly the liver, where it assists in cellular defense by facilitating the synthesis of APPs [41], [42]. Zip14 is the most highly inducible Zn transporter in liver following systemic endotoxin exposure and accounts for the majority of Zn uptake into liver tissue [16], [41], [43]. Consistent with this, our group recently discovered that Zn is transported into lung tissue and circulating monocytes via Zip8, the most closely related Zn transporter to Zip14 [44], in response to endotoxin or sepsis [23]. Importantly, Zip8-transported Zn then directly interacted with and inhibited IKKβ, a central mediator of the canonical NF-κB pathway [23]. Moreover, deficient amounts of systemic Zn resulted in less transporter-mediated uptake, decreased inhibition of IKK/NF-κB, and an exaggerated inflammatory response [22], [24]. Consistent with our findings, Zip14 knockout mice exhibited decreased Zn intake and enhanced IκBα phosphorylation in the liver following endotoxin exposure, indicative of increased NF-κB activation which also corresponded with lower tissue Zn content [43]. Collectively, these findings indicate that the specific cells and tissues are well adapted to rapidly and efficiently mobilize Zn into key cells involved in host defense and that deficits in Zn intake or its metabolism cause immune dysfunction through alteration of intracellular signaling networks.

Sepsis has profound effects on nutrient metabolism and utilization, which is further complicated by the fact that Zn interacts with a vast number of proteins. Knowing this, we utilized a global screening approach to reveal potential targets that account for the increased morbidity and mortality previously observed in Zn-deficient, septic mice [24]. Based on our long-standing interest in lung pathology relative to sepsis, we conducted genome-wide screening on this tissue. First, we observed that *Saa1* and *Saa2* were two of the most highly regulated genes that depend on Zn status. Specifically, Zn deficiency substantially up-regulated the expression of both genes in response to sepsis whereas Zn supplementation significantly decreased their expression. Consistent with this observation, network analysis of the entire database revealed that the APR was the most significantly up-regulated canonical pathway in the setting of Zn deficiency, which then directed our attention to hepatic tissue and hepatocytes. Our results from liver tissue relative to SAA expression were essentially identical to what we observed in the lung. The induction of the APR requires elaboration of early-response cytokines including IL-1, which primarily activates NF-κB, and IL-6, which predominantly activates STAT3 [45], [46], [47]. IL-6 and IL-6-mediated activation of STAT3 is crucial for the induction of APPs in hepatocytes, including but not limited to α2-macroglobulin (α2M), α1-antitrypsin, tissue inhibitor of metalloproteinases (TIMP)1, γ-fibrinogen (FGG) and SAA [6]. In support of this, we observed that Zn deficiency enhances SAA production in part through JAK-STAT3 activation. Consistent with our observation, Zn was shown to directly inhibit STAT3 activation thereby attenuating T(h)17 cell development [48]. Whether Zn directly or indirectly inhibits STAT3 within our model remains unclear; however, we did observe that Zn deficiency increased SHP1 activity. This effect could not be explained by direct inhibition of SHP1 because Zn has been shown to directly inhibit recombinant SHP1 in a cell-free system [49]. Taken together, we postulate that Zn may indirectly augment SHP1 activity by reducing reactive oxygen species (ROS) formation, which have proven inhibitory effects on SHP1 activity [21], [50], although this will require further investigation.

APPs are generally considered to be beneficial in the setting of infection, which is supported by the observation that deficiency of select APPs exacerbates infection and injury [9], [51]. Alternatively, we envision that over-production of APPs, including SAA, may also have detrimental host effects. Consistent with this notion, persistent elevation in circulating SAA levels are associated with amyloidosis and implicated in the pathogenesis of rheumatoid arthritis and Alzheimer's disease [52], [53], [54]. Increased serum SAA levels also correlate with an increased risk of cardiovascular disease [55], [56].

In summary, microarray analysis revealed that the APR and particularly SAA production are very sensitive to Zn status in the setting of sepsis. Upon further exploration, we observed that the Zn effect was mediated in part through JAK-STAT3 activation (Fig. S5). The consequences of enhanced activation manifest as over-production of SAA, resulting in an abnormally high and prolonged APR. The consequences of increased APP production manifest through altered signal transduction, thereby generating a vicious cycle of inflammation and immune dysregulation in the setting of Zn deficiency and sepsis. To our knowledge, our findings demonstrate for the first time that Zn deficiency adversely effects the APR though modulation of the JAK-STAT3 pathway in the liver; further indicating that Zn modulates multiple vital organs, cells, and signaling pathways in the host response to sepsis.

## Supporting Information

Figure S1 **Genomic analysis of mouse lung transcriptome in the combinational setting of CLP-induced sepsis and Zn status.** (**A**) The flow chart illustrates the experimental procedure: C57/B6 mice were administered a control diet (Ctrl), Zn-deficient (Zn-) diet, or a Zn-deficient diet for 18 days followed by oral Zn-supplementation (Zn+) diet for 3 more days. The three week dietary regimes were then followed by CLP and tissue harvest at 24 hrs post CLP and then microarray analysis. (**B**) A volcano plot is shown to illustrate fold-change and corresponding significance in change (p-value) between CLP and non-CLP (Ctrl) groups. The cut-off boundary is shown as a red dash line. (**C**) The volcano plot demonstrates that Zn deficiency alone did not have a substantial global influence on gene expression. (**D**) The heatmap visualization of hierarchical clustering generated from the most significant changing genes induced by CLP.
(TIF)

Figure S2 **Network analysis of mouse lung transcriptome in the combinational setting of CLP-induced sepsis and Zn status.** (**A**) Top signaling networks induced by CLP-mediated sepsis. (**B**) Top signaling networks generated from Zn-responsive genes by IPA.
(TIF)

Figure S3 **The effects of Zn deficiency (Zn-) on SAA1 production in response to IL-1 and IL-6 in HepG2 cells.** HepG2 cells were cultured in the Zn-deficient medium for 7 days. Zn was removed from fetal bovine serum (FBS) by overnight incubation with 10% Chelex (Biorad, Hercules, CA). The zinc-deficient medium was prepared with DMEM/F12 and Chelex-treated FBS. (**A**) The gene expression of SAA1. (**B**) The SAA1 levels in the supernatant of HepG2 cells (Two-way ANOVA with Bonferroni post hoc, * p<0.05).
(TIF)

Figure S4 **Zn/pyrithione inhibits STAT3 signaling at early time points after IL-1/IL-6 treatment in HepG2 cells.** The densitometry analysis is shown on the right (Two-way ANOVA with Bonferroni post hoc, * p<0.05).
(TIF)

Figure S5 **A proposed working model illustrates how Zn deficiency impacts JAK-STAT3 and NF-κB pathway, resulting in augmentation of SAA1 production.** Zn deficiency increases STAT3 activation, possibly through ROS and SHP1 modulation, leading to increased SAA1 production. SAA1 then possibly activates monocytes and other immune cells, thereby perpetuating a dysfunctional amplification loop leading to an exaggerated inflammatory response and injury.
(TIF)
