# Supplementary figures and images for: Zinc Regulates the Acute Phase Response and Serum Amyloid A Production in Response to Sepsis through JAK-STAT3 Signaling
Source: PLoS One. 2014 Apr 14;9(4):e94934. doi: 10.1371/journal.pone.0094934 (PMC3986341; doi:10.1371/journal.pone.0094934)

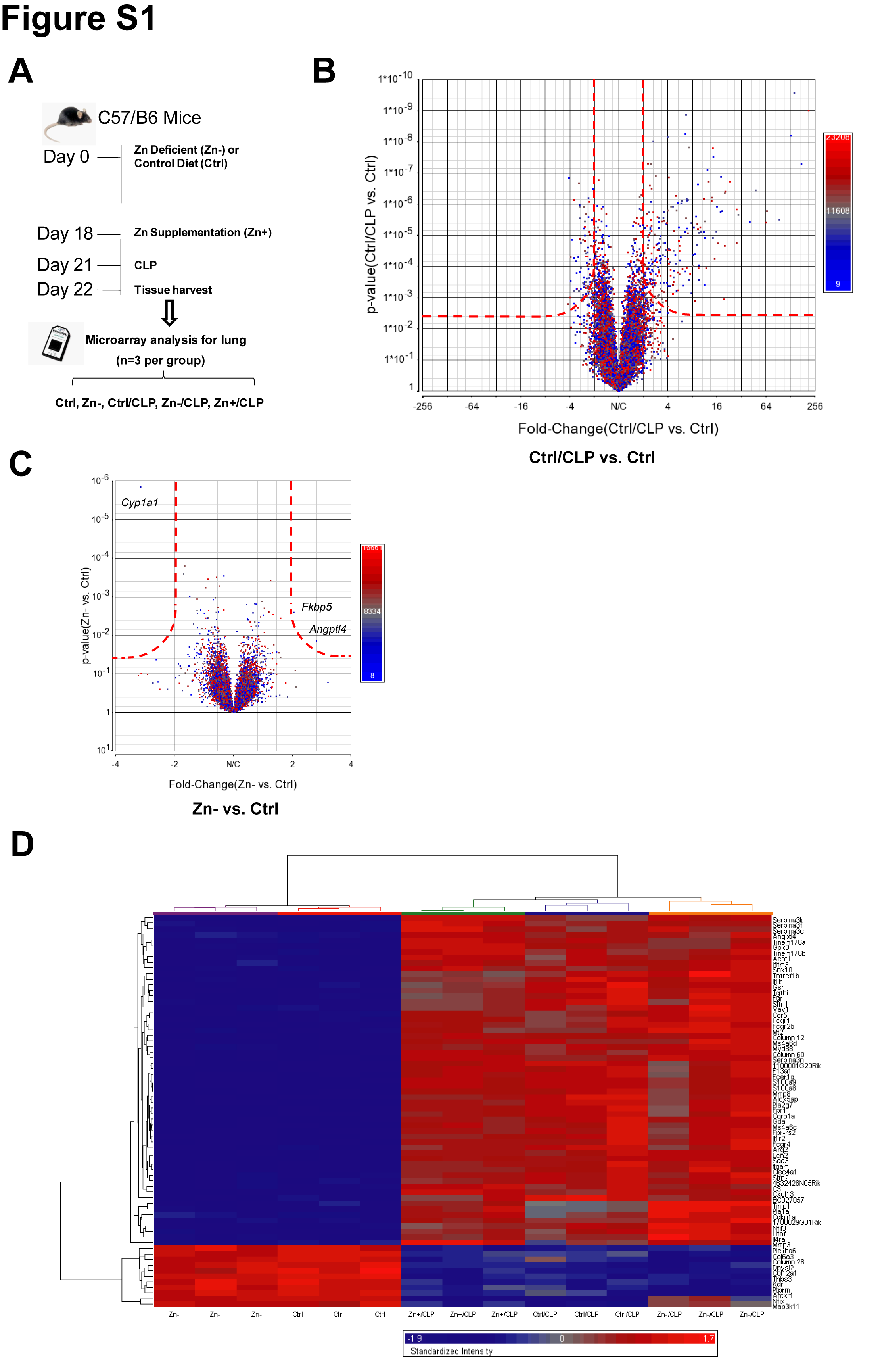

Supplement: Figure S1 — Genomic analysis of mouse lung transcriptome in the combinational setting of CLP-induced sepsis and Zn status. (A) The flow chart illustrates the experimental procedure: C57/B6 mice were administered a control diet (Ctrl), Zn-deficient (Zn-) diet, or a Zn-deficient diet for 18 days followed by oral Zn-supplementation (Zn+) diet for 3 more days. The three week dietary regimes were then followed by CLP and tissue harvest at 24 hrs post CLP and then microarray analysis. (B) A volcano plot is shown to illustrate fold-change and corresponding significance in change (p-value) between CLP and non-CLP (Ctrl) groups. The cut-off boundary is shown as a red dash line. (C) The volcano plot demonstrates that Zn deficiency alone did not have a substantial global influence on gene expression. (D) The heatmap visualization of hierarchical clustering generated from the most significant changing genes induced by CLP. (TIF) [file pone.0094934.s001.tif]

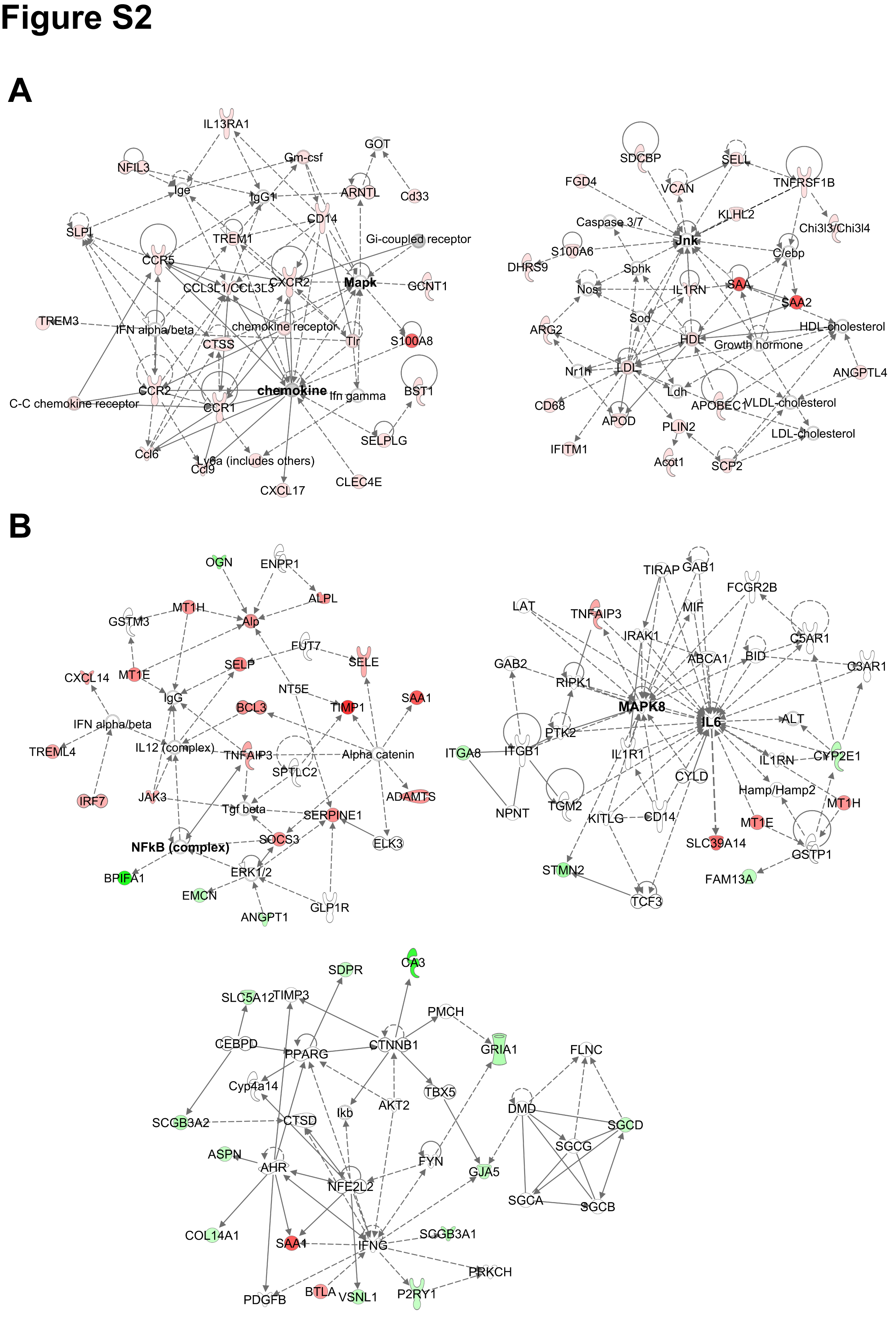

Supplement: Figure S2 — Network analysis of mouse lung transcriptome in the combinational setting of CLP-induced sepsis and Zn status. (A) Top signaling networks induced by CLP-mediated sepsis. (B) Top signaling networks generated from Zn-responsive genes by IPA. (TIF) [file pone.0094934.s002.tif]

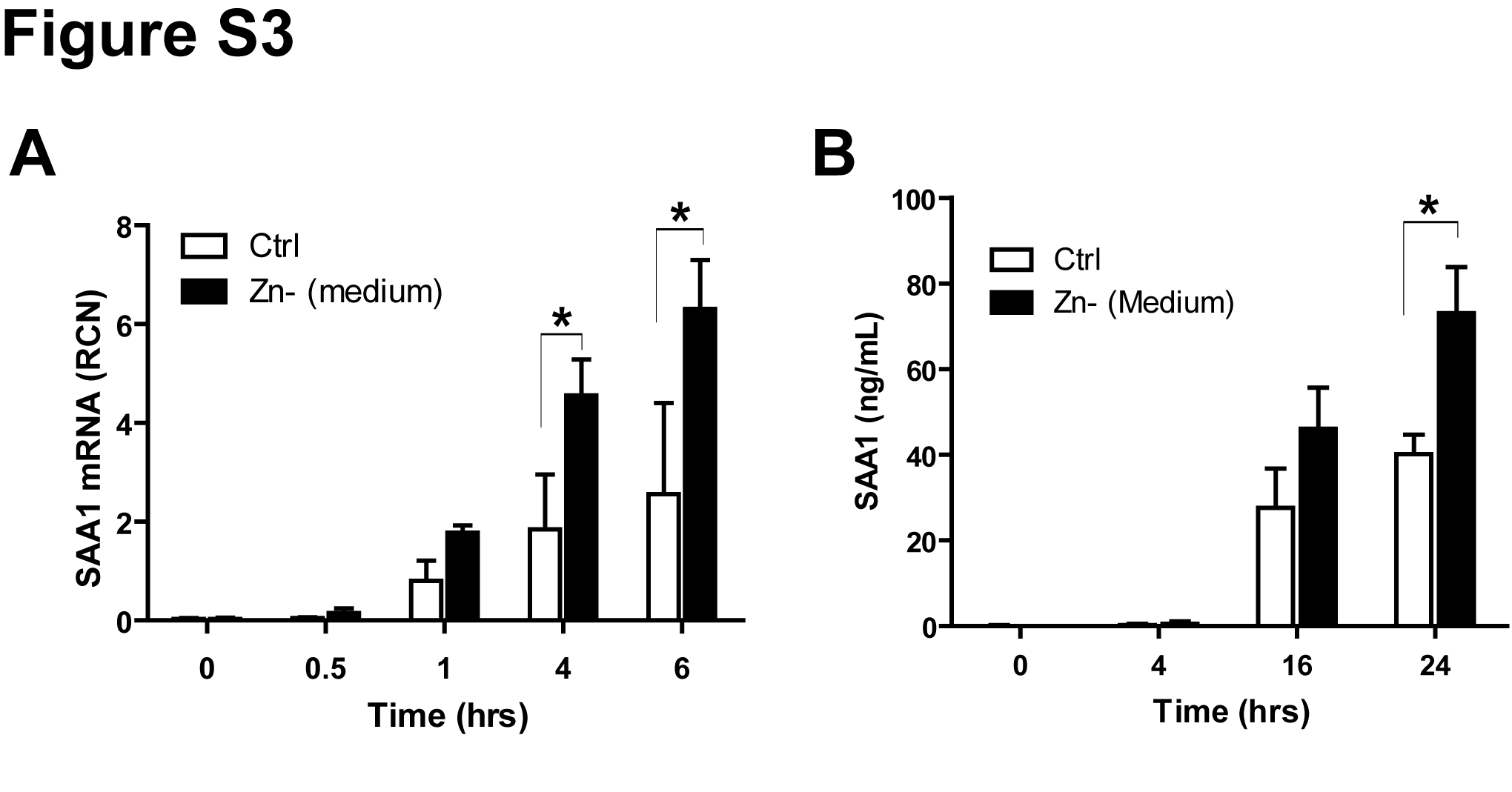

Supplement: Figure S3 — The effects of Zn deficiency (Zn-) on SAA1 production in response to IL-1 and IL-6 in HepG2 cells. HepG2 cells were cultured in the Zn-deficient medium for 7 days. Zn was removed from fetal bovine serum (FBS) by overnight incubation with 10% Chelex (Biorad, Hercules, CA). The zinc-deficient medium was prepared with DMEM/F12 and Chelex-treated FBS. (A) The gene expression of SAA1. (B) The SAA1 levels in the supernatant of HepG2 cells (Two-way ANOVA with Bonferroni post hoc, * p<0.05). (TIF) [file pone.0094934.s003.tif]

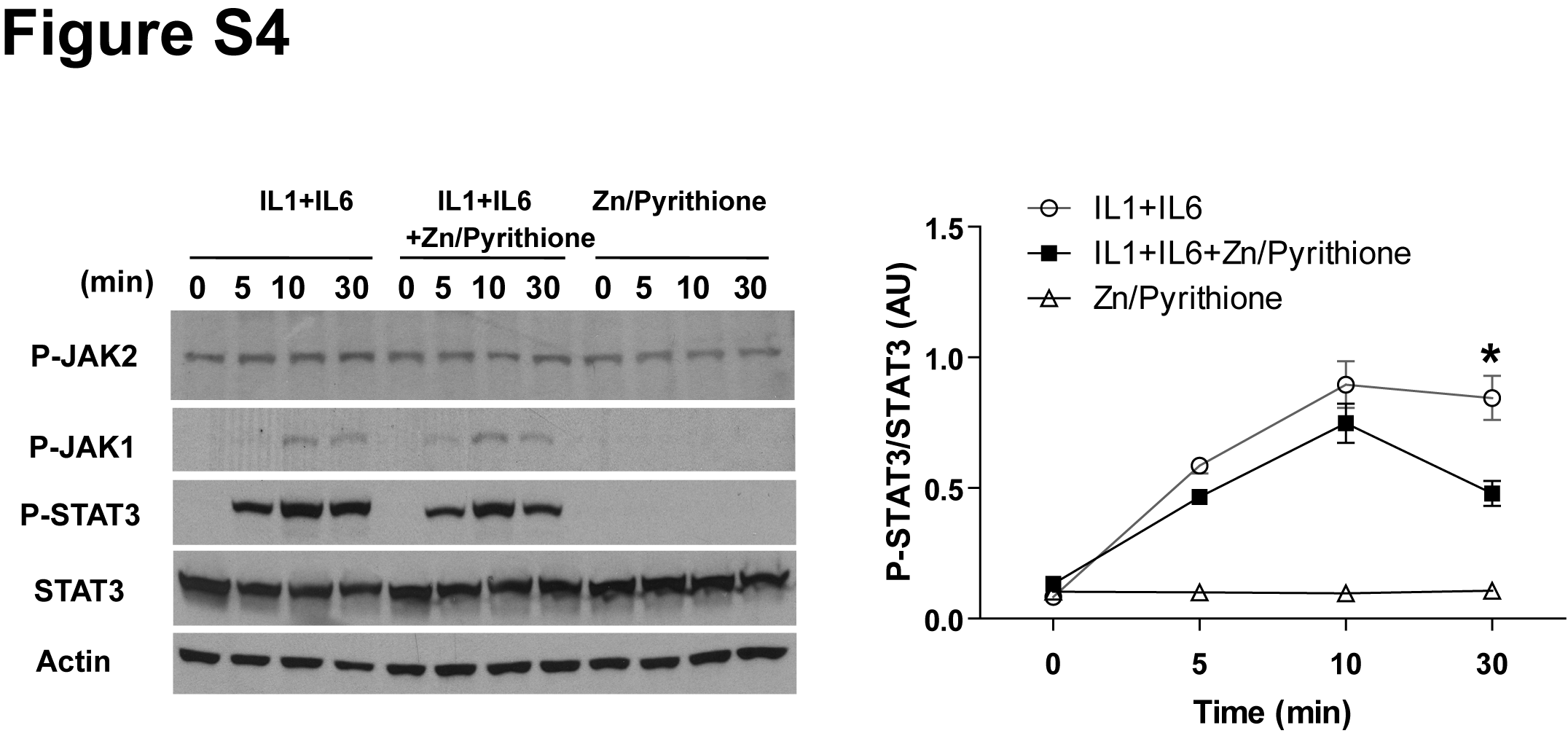

Supplement: Figure S4 — Zn/pyrithione inhibits STAT3 signaling at early time points after IL-1/IL-6 treatment in HepG2 cells. The densitometry analysis is shown on the right (Two-way ANOVA with Bonferroni post hoc, * p<0.05). (TIF) [file pone.0094934.s004.tif]

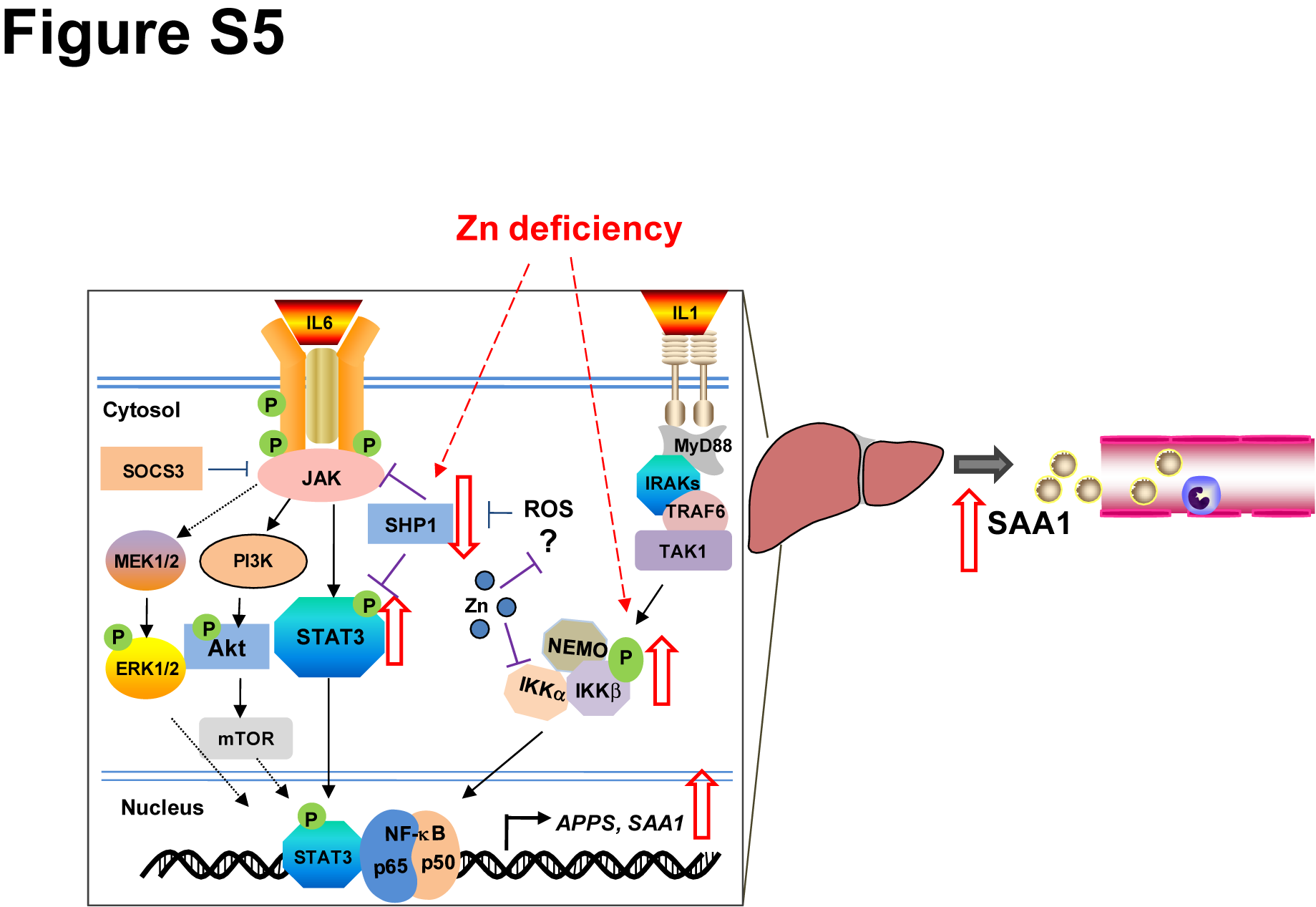

Supplement: Figure S5 — A proposed working model illustrates how Zn deficiency impacts JAK-STAT3 and NF-κB pathway, resulting in augmentation of SAA1 production. Zn deficiency increases STAT3 activation, possibly through ROS and SHP1 modulation, leading to increased SAA1 production. SAA1 then possibly activates monocytes and other immune cells, thereby perpetuating a dysfunctional amplification loop leading to an exaggerated inflammatory response and injury. (TIF) [file pone.0094934.s005.tif]
